# Coinfection of *Clostridium perfringens* and *Escherichia coli* in gas-producing perianal abscess diagnosed by 16S rDNA sequencing: a case report

**DOI:** 10.1186/s13099-021-00457-x

**Published:** 2021-10-13

**Authors:** Yang Sun, Haotian Bai, Ji Qu, Jichao Liu, Jincheng Wang, Zhenwu Du, Linlin Feng

**Affiliations:** 1 Orthopaedic Medical Center, the Second Hospital of Jilin University, Changchun, Jilin China; 2 The Engineering Research Centre of Molecular Diagnosis and Cell Treatment for Metabolic Bone Diseases of Jilin Province, Changchun, Jilin China; 3 Division of Clinical Laboratory, the Second Hospital of Jilin University, Changchun, Jilin China

**Keywords:** Gas-producing perianal abscess, 16S rDNA, DNA sequencing, Clostridial infection, Non-clostridial infection, Gas gangrene

## Abstract

**Background:**

Gas-producing perianal abscess raises the possibility of clostridial infection, with *Clostridium perfringens* being the most common causative agent, which is highly lethal if untreated timely. As the treatment of clostridial infections often differs from that of non-clostridial infections, which they may closely resemble, the importance of accurate pathogenic organism identification cannot be overemphasized. The 16S rDNA of bacteria is highly conserved within a species and among species of the same genus but demonstrates substantial variation between different species, thus making it a suitable genomic candidate for bacterial detection and identification.

**Case presentation:**

Here, we report the case of a 53-year-old patient who was admitted to the hospital for a gas-producing perianal abscess. The patient was managed with ceftizoxime and ornidazole and then received debridement and drainage at the lesion on the second day after admission. The bacterial cultures of the patient isolates from the debridement showed a coinfection of *Escherichia coli* and *Enterococcus faecium*. Although perianal redness and swelling subsided obviously after the surgery, the patient was febrile to 38.3℃ with his left upper thigh red and swollen, aggravated with tenderness and crepitus. Considering insufficient debridement and the risk of incorrect identification of pathogens, a second debridement and drainage were performed 4 days after the primary operation, and 16S rDNA sequencing of the isolates implicated *Clostridium perfringens* infection. Given the discrepancies in diagnostic results and the treatment outcomes, *Enterococcus faecium* was identified as sample contamination, and a diagnosis of coinfection of *Clostridium perfringens* and *Escherichia coli* in gas-producing perianal abscess was confirmed. The patient was then successfully treated with meropenem and vancomycin and was discharged at 27 days of admission.

**Conclusions:**

This case represents the first report of coinfection of both clostridial and non-clostridial organisms in gas-producing perianal abscess and the first case reporting the use of 16S rDNA sequencing in the diagnosis of perianal abscess. Timely pathogen identification is critical for treating gas-producing perianal abscess and an antibiotic regimen covering both aerobic and anaerobic organisms is recommended before true pathogens are identified.

**Supplementary Information:**

The online version contains supplementary material available at 10.1186/s13099-021-00457-x.

## Introduction

Perianal abscess associated with subcutaneous gas is an uncommon alarming condition that raises the possibility of soft tissue infection caused by Clostridium species, with *Clostridium perfringens* being the most common [1, 2]. The diagnosis of *Clostridium perfringens* infection is essentially clinical, as this gram-positive, spore-forming, anaerobe can cause gas gangrene (clostridial myonecrosis), which is a highly lethal, necrotizing infection of skeletal muscle and subcutaneous tissue. If untreated, the disease is 100% lethal, and it is 5 ~ 30% lethal with prompt diagnosis and appropriate treatment [2, 3]. However, considering the possibility of non-clostridial infections in gas-producing perianal abscess [4], the importance of pathogenic organism identification cannot be overemphasized, as the treatment of non-clostridial infections often differs from that of clostridial infections which they may closely resemble. Radical surgery may not be warranted, penicillin may not be the antibiotic of choice, and hyperbaric oxygen is of equivocal value [4]. Timely diagnosis followed by aggressive surgical debridement and administration of bacteria-sensitive antibiotics are keys to reducing mortality and improving prognosis of gas-producing perianal abscess [1].

In the last decade, as a result of the widespread use of PCR and DNA sequencing, 16S ribosomal DNA (rDNA) sequencing has played a pivotal role in the accurate identification of bacterial isolates in the clinic [5, 6]. The 16S rDNA gene is highly conserved within a species and among species of the same genus, but demonstrates substantial variation between different species [7]. Thus, it can be used as a new standard for the classification and identification of bacteria [8, 9]. Interrogating bacterial pathogens by this method has been extensively applied in primary research and clinical diagnosis [6, 9, 10]. Here, we report a case of initial diagnosis and treatment failure in a gas-producing perianal abscess patient coinfected by both clostridial and non-clostridial organisms, where 16S rDNA sequencing was conducted to determine the mechanism of the initial failure. This study was performed in accordance with the Helsinki Declaration of 1964 and its later amendments. Ethical approval for this study was obtained from the Clinical and Research Ethics Committee of the Second Hospital of Jilin University. Written informed consent and permission for publication of the clinical details has been obtained from the patient.

## Case presentation

A 53-year-old male was admitted to the outpatient department of the Second Hospital of Jilin University in February 2020, with swelling and pain on his left hip and proximal thigh, which he stated as having been developing over 5 days and aggravated with fever for 1 day. There was no history of trauma, and the patient was febrile (37.8 ℃). On physical examination, extensive redness and swelling spreading from the patient’s left buttocks to the thigh root could be seen at the lithotomy position, with high skin temperature and obvious tenderness; perianal connective tissue was detected protruding from the anus, and extensive soft tissue bulges were palpable on digital rectal examination, with obvious tenderness and a sense of fluctuation at the 6 o’clock position.

**Fig. 1:**
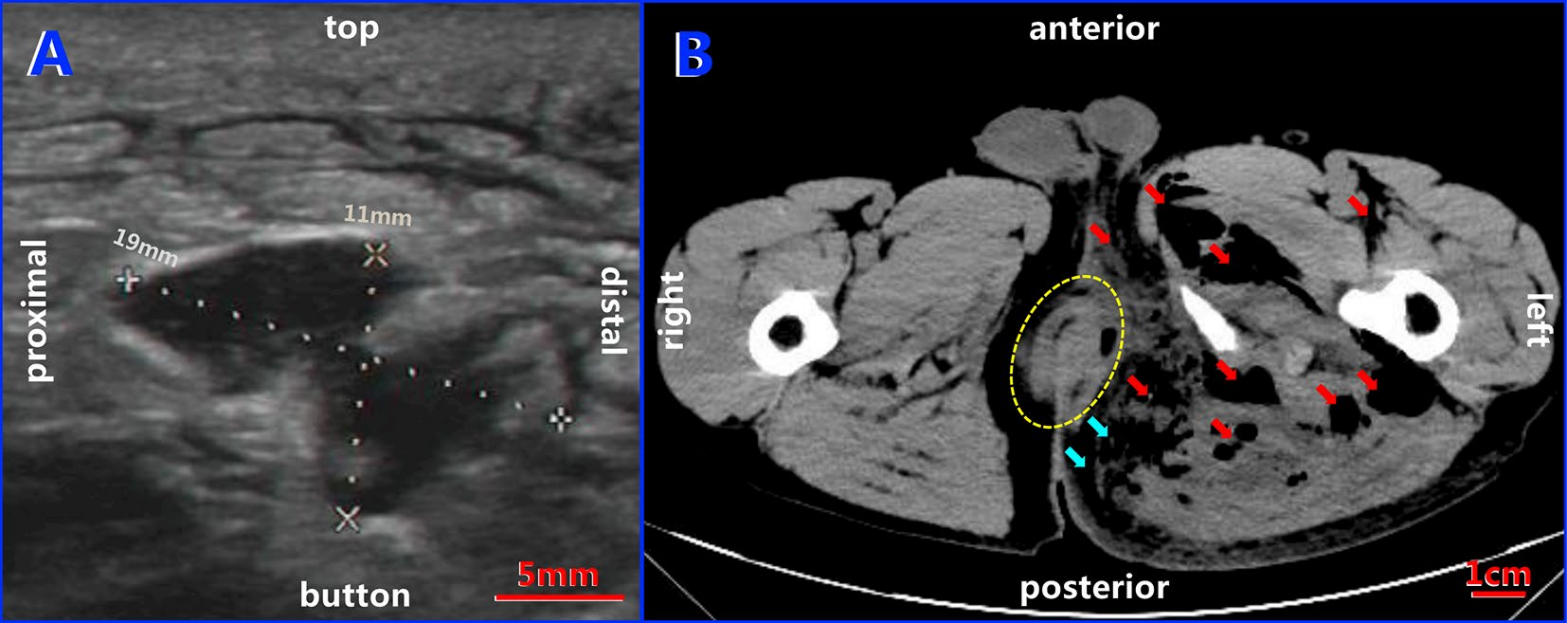
Ultrasound and CT images of the buttocks showing gas-producing perianal infection. **A** Anechoic area (1.9 cm × 1.1 cm) detected by ultrasound with a thickened subcutaneous layer. **B** Multiple liquid and gas zones (red arrows) and gas–liquid planes detected in the muscle spaces in the left hip on the transaxial CT plane

Ultrasound of the left buttocks showed the echo of abnormal soft tissue with a thickened subcutaneous layer and multiple low to anechoic stripe and flaky zones, where the most extensive area was 1.9 cm × 1.1 cm (Fig. 1A). A CT scan was obtained to further define the extent and nature of the lesion, confirming multiple patchy liquid and gas density shadows in the bilateral rectal sphincter space, bilateral ischiorectal space, left hip and left thigh root muscle space, with gas–liquid planes detected in the left hip, and the rectum wall was discontinuous at 6 o'clock (Fig. 1B). Blood examination revealed that the white blood cell count reached 16.4 × 10^9/L, with 15.7 × 10^9/L and 92.2% for neutrophils.

The patient was then diagnosed as gas-producing perianal abscess and admitted to the surgical ward with preparation for open surgery. The patient’s blood was collected for blood culture examination (5 ml cultivated for 5 days in BacT/ALERT FA culture bottles and BacT/ALERT FN culture bottles for aerobic and anaerobic bacteria detection, respectively, using BacT/ALERT 3D Microbial Detection Systems (BioMerieux Ltd., France)), and then the patient was managed with intravenous fluids and broad-spectrum antibiotics (ceftizoxime (2.0 g i.v. q12h), ornidazole (0.5 g i.v. q24h)) for empirical antibiotic therapy. On the second day after admission, the patient underwent extensive surgical debridement of the perianal abscess, revealing multiple pockets of necrotic tissue. The left hip and the proximal thigh were also debrided at multiple points where the undulations were noticeable, and then necrotic tissue was excised with drains put in place following the standard principles[11]. Over 30 ml of slightly turbid pus was released, and one intraoperative tissue specimen was sent for bacterial culture.

**Fig. 2:**
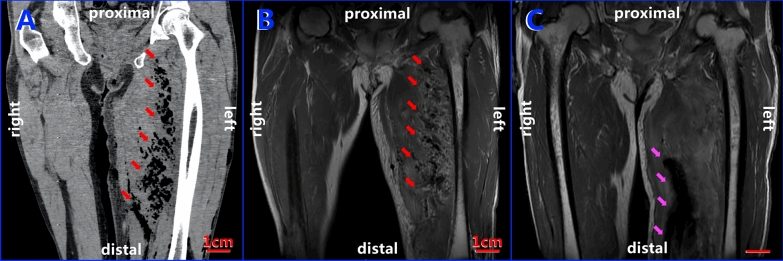
CT and MRI images showing gas-producing infection area changes in the left upper thigh. **A** CT coronal plane of the left thigh showed massive amounts of gas visible in the muscle space (red arrows); **B** corresponding to the CT image, MRI showed massive gas signals in the muscle space (red arrows); **C** MRI showed gas in the left thigh that disappeared after adjustment of the antibiotic regimen according to the 16S rDNA sequencing results, with the pink arrows pointing to the VSD equipment inserted in the lesion

Although the perianal redness and swelling subsided obviously 2 days after the procedure, the patient was still febrile (38.3℃) with his left upper thigh continuing to be red and swollen, aggravated by tenderness and crepitus. CT of the left thigh showed extensive swollen soft tissue and massive gas density visible in the muscle space extending to the knee (Fig. 2A). An MRI was obtained to further confirm that soft tissue damage and gas and fluid signals could be detected between the subcutaneous tissue and muscle spaces through the left upper thigh (Fig. 2B). Blood was collected again for a culture test.

Considering insufficient debridement and the risk of incorrect identification of pathogens, the Division of Gastrointestinal Surgery and Orthopedics performed debridement of the left upper thigh together on the 4th day after the primary operation. A large amount of inflammatory and necrotic fascia and muscle tissue was excised.VSD (Vacuum Sealing Drainage) equipment was used for closing the wound and constant drainage. Anti-infection and supportive treatment were continued after the operation, combined with 800,000 UI gentamycin in 3000 ml of 0.9% NaCl for constant irrigation. The debridement tissue was sent for bacterial culture again and a 16S rDNA sequencing test.

**Fig. 3:**
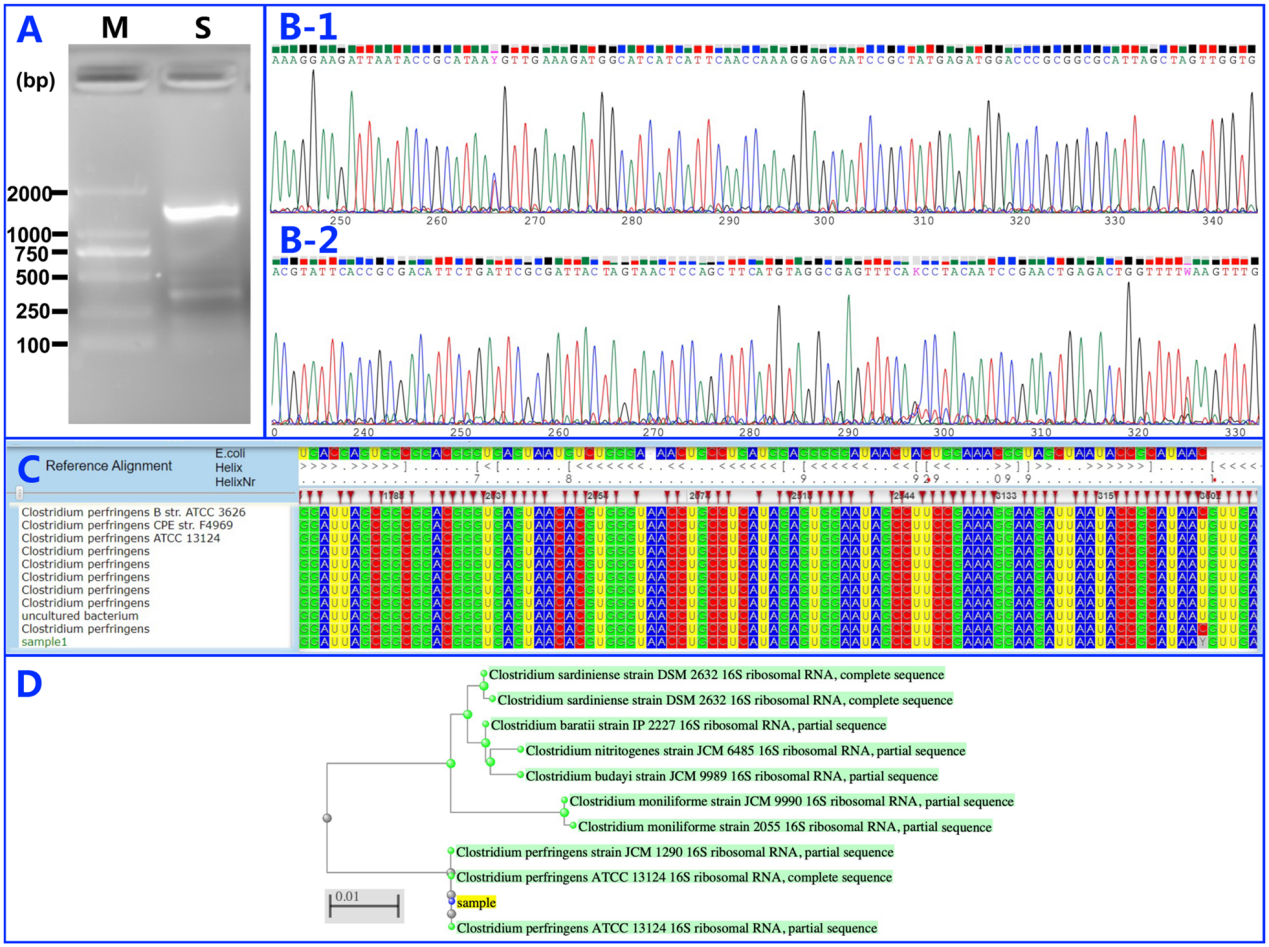
16S rDNA PCR and DNA sequencing results and identification of bacteria. **A** Results of agarose gel electrophoresis of 16S rDNA PCR products; M: DNA Marker, the size of DNA marker from top to bottom are 2000 bp, 1000 bp, 750 bp, 500 bp, 250 bp, 100 bp; S: 16S rDNA of the sample isolated from the patient. **B** Chromatogram of 16S rDNA sequencing with different sequencing primers; **B-1** forward primer; **B-2** reverse primer. **C** Silva bacterial database query results using 16S rDNA sequencing of the sample. **D** The phylogenetic tree of the 16S rDNA sequence from the sample established with the NCBI BLAST database

While the blood culture tests continued to be negative, the first sample taken intraoperatively was positive in the bacterial culture test, and later identified as coinfection of *Escherichia coli* and *Enterococcus faecium* by conventional phenotype methods using the COMPACT VITEK2 identification system (BioMerieux Ltd., France) 1 day after the second debridement [12]. Drug sensitivity tests determined that the isolated *Escherichia coli* was sensitive to amikacin, ampicillin, ampicillin-Sulbactam, aztreonam, cefazolin, cefepime, cefotetan, ceftazidime, ceftriaxone, cefuroxime, ciprofloxacin, gentamicin, imipenem, levofloxacin, meropenem, piperacillin, tobramycin, and trimethoprim, while the isolated *Enterococcus faecium* was sensitive to ampicillin, ciprofloxacin, erythromycin, high-level gentamicin, levofloxacin, linezolid, penicillin-G, tetracycline, tigecycline, and vancomycin. Although the specimen from the second surgery was negative in the bacterial culture test, the bacterial 16S rDNA from the patient sample was detected by PCR amplification with the 16S rDNA Bacterial Identification PCR Kit (Code No. RR176, TaKaRa, China). The sequence of the forward primer was 5′-GAGCGGATAACAATTTCACACAGG-3′, and the sequence of the reverse primer was 5′-CGCCAGGGTTTTCCCAGTCACGAC-3′. PCR results showed that there was an apparent PCR product of approximately 1600 bp representing the full-length 16S rDNA found in agarose gel (Fig. 3A). To clarify the classification of bacteria, DNA sequencing of this PCR amplicon was performed by the Sanger sequencing method [13]. The chromatogram of 16S rDNA sequencing with different sequencing primers showed a single peak, indicating that one bacterial 16S rDNA fragment was present among PCR products (Fig. 3B). The bacteria were identified by searching and comparing the 16S rDNA sequences (see Additional file 1) using the Silva database (https://www.arb-silva.de/aligner), which indicated that the bacteria have a 99% identity classification for *Clostridium perfringens* (Fig. 3C), and the phylogenetic tree was established by using the Basic Local Alignment Search Tool (BLAST) offered by the National Centre for Biotechnology Information (NCBI) database (https://blast.ncbi.nlm.nih.gov) and confirmed that the isolate was most closely related to *Clostridium perfringens* (Fig. 3D).

Due to the detection of *Clostridium perfringens* by 16S rDNA sequencing, the antibiotic regimen was then adjusted according to the results of drug sensitivity tests following bacterial cultures and DNA sequencing tests: meropenem (1 g i.v. q8h) and vancomycin (1 g i.v. q12h). The pain, redness, and swelling of the perianal area and left upper thigh continued to be relieved by this regimen. MRI indicated that although the soft tissue of the left thigh was obviously thickened, no obvious abnormal signal between/in the muscle and bone tissue was detected (Fig. 2C). Twelve days after the second debridement, another operation was performed again to remove the VSD equipment and close the wound. The blood examination taken on the 3rd and 5th days postoperatively showed no inflammation and the anti-infective treatment was then stopped. After another 3 days of close observation, the patient was discharged at 27 days of admission, and recovered well without adverse complaints at the time of 1-month follow-up.

## Discussion and conclusions

Although clostridial infection is the most likely reason for gas-producing perianal abscess, non-clostridial infections cannot be overlooked[4, 14]. The underlying microbiology of this patient’s aggressive perianal infection was first investigated clinically by conventional modalities. While the blood cultures remained negative, the first culture of debridement material indicated coinfection of *Escherichia coli*, which is one of the most common aerobic organisms resulting in perianal abscess [15], and *Enterococcus faecium*, leading to a provisional diagnosis of non-clostridial gas-producing perianal infection [1, 4]. However, in contrast to the negative result of the second culture of debridement tissue, molecular testing by 16S rDNA gene sequencing was positive for an entirely different organism, confidently identified as *Clostridium perfringens*. Meanwhile, considering that the initial antibiotic regimen was invalid against *Enterococcus faecium*, which originates in the gastrointestinal tract [16], the negative result of the second culture most likely indicated the possibility of sample contamination during the debridement and material collection procedures. When taking into account the results of the drug sensitivity test performed on the isolated *Escherichia coli* and the short-term clinical symptom improvement after the first debridement, the negative results of the second culture and 16S rDNA gene sequencing to detect *Escherichia coli* likely indicated the elimination of this pathogen by the initial antibiotic treatment and debridement surgery. Given these discrepancies in diagnostic results, although the possibility that *Escherichia coli* was also a contaminating bacteria could not be completely ruled out, a diagnosis of coinfection by *Clostridium perfringens* and *Escherichia coli* was favored by the inpatient team due to the severity of the infection and because it penetrated through tissue spaces with myonecrosis [2], leading to election of an antimicrobial therapy expected to cover both aerobic and anaerobic organisms broadly. To the knowledge of the authors, coinfection by clostridial and non-clostridial organisms in the gas-producing perianal abscess has never been reported.

The 16S rDNA sequence represents a suitable genomic candidate for bacterial detection and identification, as the sequences of some regions of the 16S rDNA are homologous in all bacteria, while other regions of this gene show considerable variation between species [7, 17]. For pathogen identification, 16S rDNA sequencing is particularly important in the case of bacteria with unusual phenotypic profiles, rare bacteria, slow-growing bacteria, uncultivable bacteria and culture-negative infections [5]. Here, 16S rDNA sequencing, for the first time, was employed to more directly detect the bacteria comprising the patient’s perianal infection by sequencing bacterial genes amplified from patient material. In disagreement with conventional bacterial culture results, the 16S rDNA sequencing alignment results indicated *Clostridium perfringens*. Given this molecular finding, the failure to detect *Escherichia coli* by the second culture most likely reflects the destruction of the organism in response to antibiotic therapy prior to sample collection, although the sensitivity of 16S rDNA sequencing might be superior to that of culture in cases with antibiotic exposure [18, 19]. Meanwhile, the negative culture results of *Clostridium perfringens* may reflect the selective lysis of the pathogen resulting from exposure of the sample to air during sample processing, although patient specimens that arrived at the laboratory were appropriately transported for anaerobic organisms, or the strain failed to grow outside dedicated anaerobic enrichment medium [10]. Thus, disparate laboratory diagnoses were confirmed independently and amalgamated into a single, more comprehensive result showing coinfection of *Clostridium perfringens* and *Escherichia coli.*

Although the culture of debridement material and DNA sequencing modalities indicated polymicrobial infection, it is essential to note that there could be some other conventional methods that are helpful for identifying the organisms. Gram staining of Clostridium will show large gram-positive rods, with a paucity of leukocytes (as is typical of anaerobic infections) [2], while *Escherichia coli* shows gram-negative rods, and *Enterococcus faecium* shows gram-positive cocci [20, 21]. In this case, the medical group did not perform Gram staining due to the clinical laboratory chaos caused by the coronavirus disease 2019 (COVID-19) outbreak at the beginning of last year [22, 23]. However, because cellular morphology and arrangement are insufficient for differentiating the species directly, Gram strains, although efficient and compensatory, are sometimes the only distinguishing characteristic while cultures and subsequent drug sensitivity experiments are still the conventional critical tests for early and accurate pathogen diagnosis. Considering the risk of clostridial infection and the possibility of non-clostridial infection in gas-producing perianal abscess, an antimicrobial therapy covering both aerobic and anaerobic bacteria was used by the authors and should be recommended in such cases before an accurate diagnosis is made. This may also help to explain why the clostridial perianal infection was controlled effectively without hyperbaric oxygenation, which is commonly suggested in the management of gas gangrene [2, 24].

This case represents the first report of coinfection of both clostridial and non-clostridial organisms in gas-producing perianal abscess and highlights the importance of using 16S rDNA sequencing to make an accurate diagnosis in patients with gas-producing perianal abscess. Although Gram staining and cultures are still commonly used for characterizing and differentiating species, to improve the sensitivity of pathological organism detection, especially for anaerobes, 16S rDNA sequencing should be considered to ensure timely diagnosis and treatment. Before true pathogens are identified, an antibiotic regimen covering both aerobic and anaerobic organisms is recommended for treating gas-producing perianal abscess.

## Supplementary Information

**Additional file 1: Figure S1.** Partial 16S rDNA gene sequence of the patient’s isolate. A: gene sequence amplified from the forward primer. B: gene sequence amplified from the reverse primer.

## Data Availability

All data generated or analyzed during this study are included in this published article and its supplementary information files. The dataset (partial 16S rDNA gene sequence of the patient’s isolate) supporting the conclusions of this article is available in the Figshare repository at: https://doi.org/10.6084/m9.figshare.14934108.v1

## Abbreviations

rDNA: Ribosomal DNA
CT: Computed tomography
VSD: Vacuum sealing drainage
BLAST: Basic Local Alignment Search Tool
NCBI: National Centre for Biotechnology Information
COVID-19: Corona Virus Disease 2019

## References

[1] Raskov HH, Kirkegaard P (1985). Gas-producing perianal infection. Acta Chir Scand.

[2] Buboltz JB, Murphy-Lavoie HM. Gas Gangrene. StatPearls. Treasure Island (FL): StatPearls Publishing Copyright © 2021, StatPearls Publishing LLC.; 2021. 30725715

[3] Leiblein M, Wagner N, Adam EH, Frank J, Marzi I, Nau C (2020). Clostridial Gas Gangrene - a rare but deadly infection: case series and comparison to other necrotizing soft tissue infections. Orthop Surg.

[4] Brightmore T (1972). Perianal gas-producing infection of non-clostridial origin. Br J Surg.

[5] Woo PC, Lau SK, Teng JL, Tse H, Yuen KY (2008). Then and now: use of 16S rDNA gene sequencing for bacterial identification and discovery of novel bacteria in clinical microbiology laboratories. Clin Microbiol Infect.

[6] De R, Mukhopadhyay AK, Dutta S (2020). Metagenomic analysis of gut microbiome and resistome of diarrheal fecal samples from Kolkata, India, reveals the core and variable microbiota including signatures of microbial dark matter. Gut Pathogens.

[7] Neefs JM, Van de Peer Y, De Rijk P, Goris A, De Wachter R (1991). Compilation of small ribosomal subunit RNA sequences. Nucleic Acids Res.

[8] Woo PC, Lau SK, Chan KM, Fung AM, Tang BS, Yuen KY (2005). Clostridium bacteraemia characterised by 16S ribosomal RNA gene sequencing. J Clin Pathol.

[9] Wu J, Gan T, Zhang Y, Xia G, Deng S, Lv X, Zhang B, Lv B (2020). The prophylactic effects of BIFICO on the antibiotic-induced gut dysbiosis and gut microbiota. Gut Pathogens.

[10] Salipante SJ, Hoogestraat DR, Abbott AN, SenGupta DJ, Cummings LA, Butler-Wu SM, Stephens K, Cookson BT, Hoffman NG (2014). Coinfection of Fusobacterium nucleatum and Actinomyces israelii in mastoiditis diagnosed by next-generation DNA sequencing. J Clin Microbiol.

[11] Sahnan K, Adegbola SO, Tozer PJ, Watfah J, Phillips RK (2017). Perianal abscess. BMJ..

[12] Bemer P, Juvin ME, Le Gargasson G, Drugeon H, Reynaud A, Corvec S (2010). Correlation between the VITEK2 system and cefoxitin disk diffusion for the daily detection of oxacillin resistance in a large number of clinical Staphylococcus aureus isolates. Eur J Clin Microbiol Infect Dis.

[13] Han XY, Golshan MA, Bowman CJ (2020). Concurrent Cultivation of Mycobacterium avium and Mycobacterium intracellulare Identified by a Single Sanger Sequencing of the 16S Gene. J Clin Microbiol..

[14] Adamo K, Sandblom G, Brännström F, Strigård K (2016). Prevalence and recurrence rate of perianal abscess–a population-based study, Sweden 1997–2009. Int J Colorectal Dis.

[15] Liu CK, Liu CP, Leung CH, Sun FJ (2011). Clinical and microbiological analysis of adult perianal abscess. J Microbiol Immunol Infect.

[16] Caballero S, Kim S, Carter RA, Leiner IM, Sušac B, Miller L, Kim GJ, Ling L, Pamer EG (2017). Cooperating commensals restore colonization resistance to vancomycin-resistant *Enterococcus faecium*. Cell Host Microbe.

[17] Gurtler V, Wilson VA, Mayall BC (1991). Classification of medically important clostridia using restriction endonuclease site differences of PCR-amplified 16S rDNA. J Gen Microbiol.

[18] Huang YJ, Kim E, Cox MJ, Brodie EL, Brown R, Wiener-Kronish JP, Lynch SV (2010). A persistent and diverse airway microbiota present during chronic obstructive pulmonary disease exacerbations. OMICS.

[19] Coiffier G, David C, Gauthier P, Le Bars H, Guggenbuhl P, Jolivet-Gougeon A, Albert JD (2019). Broad-range 16 s rDNA PCR in synovial fluid does not improve the diagnostic performance of septic arthritis in native joints in adults: cross-sectional single-center study in 95 patients. Clin Rheumatol.

[20] Lerner AM (1980). The gram-negative bacillary pneumonias. Dis Mon.

[21] Facklam R, Hollis D, Collins MD (1989). Identification of gram-positive coccal and coccobacillary vancomycin-resistant bacteria. J Clin Microbiol.

[22] Jawhara S (2020). How to boost the immune defence prior to respiratory virus infections with the special focus on coronavirus infections. Gut Pathogens.

[23] Khosrawipour V, Lau H, Khosrawipour T, Kocbach P, Ichii H, Bania J, Mikolajczyk A (2020). Failure in initial stage containment of global COVID-19 epicenters. J Med Virol.

[24] Stevens DL, Bryant AE (2017). Necrotizing soft-tissue infections. N Engl J Med.

