# Supplementary figures and images for: Coinfection of Clostridium perfringens and Escherichia coli in gas-producing perianal abscess diagnosed by 16S rDNA sequencing: a case report
Source: Gut Pathog. 2021 Oct 13;13:61. doi: 10.1186/s13099-021-00457-x (PMC8511849; doi:10.1186/s13099-021-00457-x)

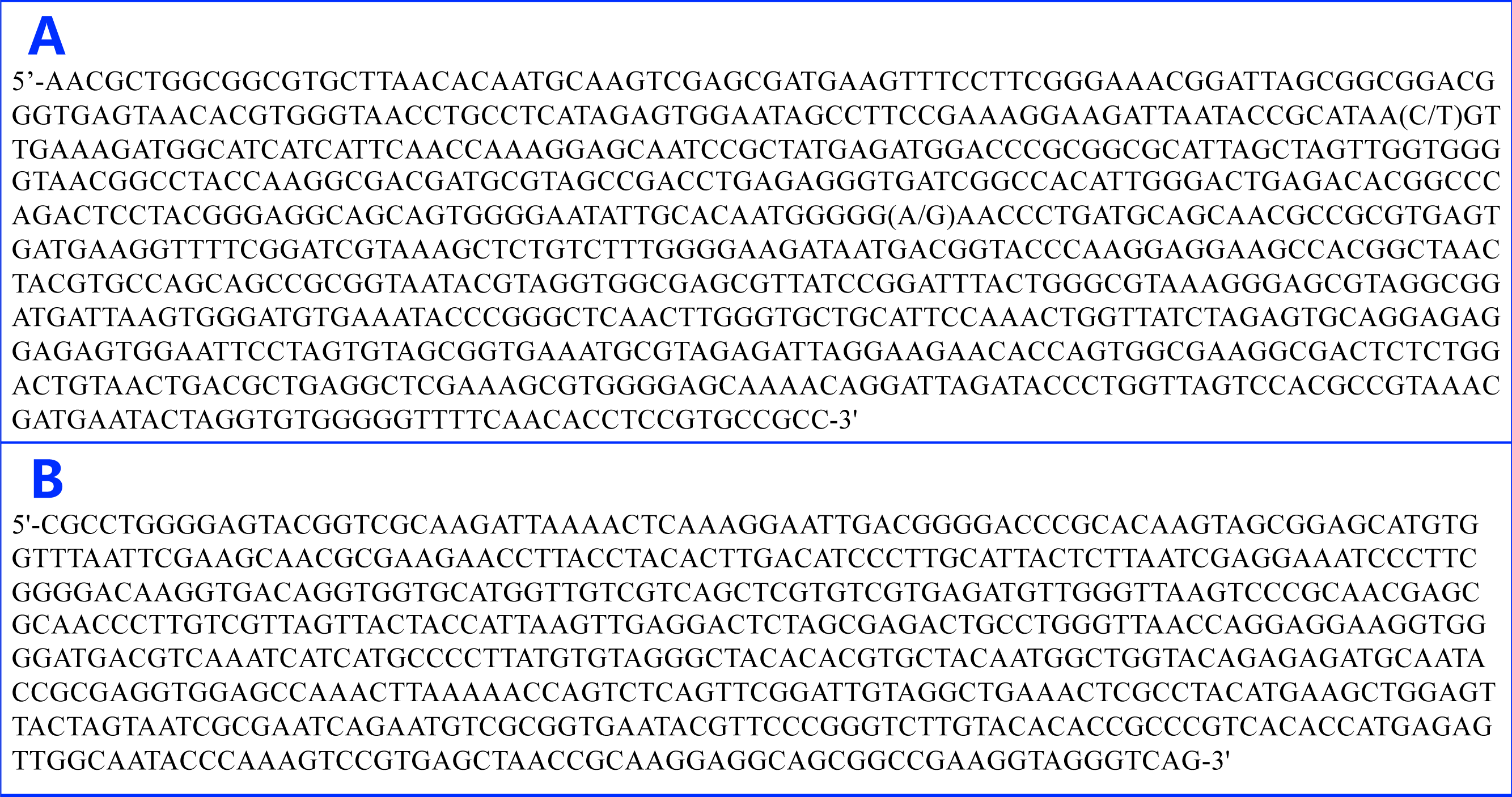

Supplement: Supplementary file 1 — Additional file 1: Figure S1. Partial 16S rDNA gene sequence of the patient’s isolate. A: gene sequence amplified from the forward primer. B: gene sequence amplified from the reverse primer. [file 13099_2021_457_MOESM1_ESM.tif]
